# Validation of Prediction Models for Critical Care Outcomes Using Natural Language Processing of Electronic Health Record Data

**DOI:** 10.1001/jamanetworkopen.2018.5097

**Published:** 2018-12-21

**Authors:** Ben J. Marafino, Miran Park, Jason M. Davies, Robert Thombley, Harold S. Luft, David C. Sing, Dhruv S. Kazi, Colette DeJong, W. John Boscardin, Mitzi L. Dean, R. Adams Dudley

**Affiliations:** 1 Philip R. Lee Institute for Health Policy Studies, School of Medicine, University of California, San Francisco; 2 Center for Healthcare Value, University of California, San Francisco; 3 currently with Biomedical Informatics Training Program, Stanford University School of Medicine, Stanford, California; 4 Department of Neurological Surgery, University of California, San Francisco; 5 Departments of Neurosurgery and Biomedical Informatics, University of Buffalo, Buffalo, New York; 6 Palo Alto Medical Foundation Research Institute, Palo Alto, California; 7 Department of Orthopedic Surgery, Boston Medical Center, Boston, Massachusetts; 8 Division of Cardiology, Zuckerberg San Francisco General Hospital, San Francisco, California; 9 Department of Epidemiology and Biostatistics, University of California, San Francisco; 10 Department of Medicine, University of California, San Francisco

## Abstract

**Importance:**

Accurate prediction of outcomes among patients in intensive care units (ICUs) is important for clinical research and monitoring care quality. Most existing prediction models do not take full advantage of the electronic health record, using only the single worst value of laboratory tests and vital signs and largely ignoring information present in free-text notes. Whether capturing more of the available data and applying machine learning and natural language processing (NLP) can improve and automate the prediction of outcomes among patients in the ICU remains unknown.

**Objectives:**

To evaluate the change in power for a mortality prediction model among patients in the ICU achieved by incorporating measures of clinical trajectory together with NLP of clinical text and to assess the generalizability of this approach.

**Design, Setting, and Participants:**

This retrospective cohort study included 101 196 patients with a first-time admission to the ICU and a length of stay of at least 4 hours. Twenty ICUs at 2 academic medical centers (University of California, San Francisco [UCSF], and Beth Israel Deaconess Medical Center [BIDMC], Boston, Massachusetts) and 1 community hospital (Mills-Peninsula Medical Center [MPMC], Burlingame, California) contributed data from January 1, 2001, through June 1, 2017. Data were analyzed from July 1, 2017, through August 1, 2018.

**Main Outcomes and Measures:**

In-hospital mortality and model discrimination as assessed by the area under the receiver operating characteristic curve (AUC) and model calibration as assessed by the modified Hosmer-Lemeshow statistic.

**Results:**

Among 101 196 patients included in the analysis, 51.3% (n = 51 899) were male, with a mean (SD) age of 61.3 (17.1) years; their in-hospital mortality rate was 10.4% (n = 10 505). A baseline model using only the highest and lowest observed values for each laboratory test result or vital sign achieved a cross-validated AUC of 0.831 (95% CI, 0.830-0.832). In contrast, that model augmented with measures of clinical trajectory achieved an AUC of 0.899 (95% CI, 0.896-0.902; *P* < .001 for AUC difference). Further augmenting this model with NLP-derived terms associated with mortality further increased the AUC to 0.922 (95% CI, 0.916-0.924; *P* < .001). These NLP-derived terms were associated with improved model performance even when applied across sites (AUC difference for UCSF: 0.077 to 0.021; AUC difference for MPMC: 0.071 to 0.051; AUC difference for BIDMC: 0.035 to 0.043; *P* < .001) when augmenting with NLP at each site.

**Conclusions and Relevance:**

Intensive care unit mortality prediction models incorporating measures of clinical trajectory and NLP-derived terms yielded excellent predictive performance and generalized well in this sample of hospitals. The role of these automated algorithms, particularly those using unstructured data from notes and other sources, in clinical research and quality improvement seems to merit additional investigation.

## Key Points

**Question:**

Can a prediction model for mortality in the intensive care unit be improved by using more laboratory values, vital signs, and clinical text in electronic health records?

**Findings:**

In this cohort study of 101 196 patients in the intensive care unit, a machine learning–based model using all available measurements of vital signs and laboratory values, plus clinical text, exhibited good calibration and discrimination in predicting in-hospital mortality, yielding an area under the receiver operating characteristic curve of 0.922.

**Meaning:**

Applying methods from machine learning and natural language processing to information already routinely collected in electronic health records, including laboratory test results, vital signs, and clinical free-text notes, significantly improves a prediction model for mortality in the intensive care unit compared with approaches that use only the most abnormal vital sign and laboratory values.

## Teaser

This cohort study evaluates the change in power for a mortality predictive model by incorporating measures of clinical trajectory and natural language processing of clinical text from the electronic health records of patients in the intensive care unit.

## Introduction

Patients in intensive care units (ICUs) vary markedly in terms of their likelihood of survival. Models that predict mortality accurately and that can be easily automated can foster internal quality improvement, cross-institutional comparisons, and clinical research in the ICU.^1,2,3,4,5^

Most current ICU mortality modeling methods use a small fraction of the data available on a patient, primarily the single most abnormal value of laboratory test results and vital signs, and none of the clinical text. Developed before electronic health records (EHRs) were widely adopted, these models relied on manual data abstraction and thus had a compelling rationale to limit the data collected. For example, a manual Acute Physiology and Chronic Health Evaluation (APACHE) medical record review by a trained nurse takes an average of 30 minutes per patient.^6^ Although most of this process can be automated with EHRs,^7,8,9^ this approach still predominates in current modeling paradigms. This process has clear limitations; for example, a brief elevation in heart rate and a sustained tachyarrhythmia are treated similarly, and a transient reduction in the Glasgow Coma Scale score resulting from acute alcohol intoxication receives similar treatment as sustained deterioration from a stroke (eFigure 1 in the Supplement). The increasing adoption of EHRs allows all values of a variable, such as the Glasgow Coma Scale score, to be used in such models, and thereby allows patients’ clinical trajectories to be assessed. Doing so may yield more accurate mortality prediction models, but to our knowledge this hypothesis has not been tested to date.

Another way to take advantage of EHR data is to process the information present in text notes, including results of the physical examination and assessment. Natural language processing (NLP) methods enable terms in notes, such as *sepsis*, *pupils fixed*, and *coagulopathy*, to be included in models.^10^ However, the possible gains in predictive power afforded by including such terms are unknown, as is the generalizability of models using this approach. Namely, whether between-institution differences in documentation patterns could limit how well models incorporating text may perform at any single institution remains unclear.

Using EHR data from 20 ICUs at 3 hospitals—2 academic medical centers and 1 community hospital—we developed and validated ICU mortality prediction models incorporating measures of clinical trajectory derived from all data points associated with a set of laboratory test results and vital signs. We also used NLP to incorporate words from notes into these models. Finally, we assessed the external validity of these models when developed at each hospital in our study and then validated on data from other hospitals.

## Methods

### Data Sets

In this cohort study, the data used were routinely collected in the process of care delivered in 20 ICUs across 3 sites from January 1, 2001, through June 1, 2017. The sites included the University of California, San Francisco (UCSF) and Beth Israel Deaconess Medical Center (BIDMC), Boston, Massachusetts,^11^ academic, tertiary care hospitals and Mills-Peninsula Medical Center (MPMC), Burlingame, California, a 403-bed community hospital. Adult patients (aged ≥18 years) in medical, surgical, general medical/surgical, cardiac, and neurologic ICUs were selected. Both UCSF and MPMC used the same EHR system (Epic Systems Corp), whereas BIDMC data were derived from an EHR-based research database.^11^ We selected patients with an ICU stay of at least 4 hours and used only the first ICU admission during the study period for each patient. Patient demographics and discharge disposition were determined from hospital census and admit-discharge-transfer data. This study was approved by the Committee on Human Research at UCSF and the Sutter Health institutional review board, which waived the need for informed consent for the use of deidentified data. Reporting followed the Transparent Reporting of a Multivariable Prediction Model for Individual Prognosis or Diagnosis (TRIPOD) reporting guideline.^12^

We chose a set of vital signs and laboratory tests (eTable 1 in the Supplement) used in existing mortality models, including the APACHE IV, the Mortality Probability Admission Model III,^13^ and the Simplified Acute Physiology Score III.^14,15,16^ We then developed algorithms to capture from the data all observations of these variables from the first 24 hours of the ICU admission, as well as all notes written during this period, which were not deidentified, except those from BIDMC.

### Model Development

We developed clinical trajectory models leveraging serial data points for each predictor variable (eFigure 1 in the Supplement). These models rely on feature engineering algorithms,^17^ commonly used in machine learning practice, that process all available observations in the first 24 hours for each laboratory test result and vital sign and derive measures of clinical trajectory (eTable 2 in the Supplement). We imputed values of these measures for patients having no observations of a test or vital sign using the median nonmissing value of each derived measure of trajectory, which we preferred to multiple imputation methods, owing to computational and implementational considerations, and to the *k*-nearest neighbor imputation, which gave comparable performance.

We also sought to enrich these clinical trajectory models with information from clinical notes. First, we filtered notes to include only the 1000 most frequent terms occurring at each site. Then, we created a note set for each patient by combining all notes from 24 hours after ICU admission. We used the term frequency–inverse document frequency algorithm^18^ to weigh the frequency of each term in these note sets—such as *sepsis* or *respiratory acidosis* or *not septic*—relative to the proportion of note sets in which it appears. Thus, more rare terms, such as *transfusion* or *ECMO* (extracorporeal circulation membrane oxygenation), are assigned greater weight compared with more common terms, such as *plan*, which appear in nearly every progress note. Furthermore, to address copying and pasting in notes, we used a sublinear form of term frequency that took the logarithm of the frequency of a term in a note set, thus yielding diminishing returns for these weights. These weights were incorporated directly as predictors associated with mortality into our models.

We used logistic regression to model the association between in-hospital mortality and the measures of clinical trajectory with or without NLP terms. To facilitate interpretation and to guard against overfitting, predictors were treated as linear for all models. To increase predictive performance and further reduce the risk of overfitting, we constrained the complexity of the models using an *L_2_* (or ridge) penalty to control the sizes of the coefficients for the predictors.^19,20^

Overall, our approach thus differs from existing models in the following 2 ways: (1) by using information present across all observations of each laboratory test or vital sign to build measures of clinical trajectory; and (2) by adding variables derived via NLP. To assess the relative contribution of each step to predictive power relative to a baseline, we built 3 models using data from all 3 participating hospitals. The baseline model used only the maximum and minimum values of each laboratory test result or vital sign as a surrogate for models using only the most abnormal values. The second clinical trajectory–augmented model incorporated measures of variability and clinical trajectory calculated from all observations of these tests and vital signs (eTable 2 in the Supplement). Finally, the third model combined these clinical trajectory variables with those derived via NLP of notes.

### Model Validation

We undertook 2 strategies to validate these 3 models. First, for each of the 3 approaches, we built 3 separate site-specific models reflecting the case mix and documentation patterns at each site. To assess the external validity of each approach, particularly that of using terms derived via NLP, these site-specific models were then tested at each of the 2 other participating institutions.

Second, because most validation studies of ICU models pool data from institutions to attempt to build a model that generalizes well across institutions, we similarly pooled data from all 3 hospitals in our study and performed nested 10-fold cross-validation^21,22^ to obtain overall estimates of discrimination and assess the relative contribution of each of the approaches above to overall model performance. Cross-validation was used over split-sample validation, because in the context of the bias-variance trade-off,^20^ it yields performance estimates with lower variance; using nested cross-validation likewise reduces the bias of these cross-validation estimates.^21,22^

We assessed model performance by computing the area under the receiver operating characteristic curve (AUC)^23^ to evaluate discrimination for each model. Estimates of model discrimination are reported as the mean AUC across all repetitions of cross-validation. We computed modified Hosmer-Lemeshow test statistics^24^ to assess calibration and considered a model well calibrated if *P* > .05 for the test statistic.^25^ In addition, we also computed area under the precision-recall curve (AUPRC)^26^ for each of these 3 models.

Finally, we also considered that including these additional variables could introduce bias by associating mortality with variables measured just before death for those patients who survived less than 24 hours. For example, terms derived from notes could include *expired* or *CMO* (comfort measures only), which would predict death with certainty, potentially biasing a model as it learns to associate these terms with mortality and thus crowding out other predictors. Therefore, we conducted a sensitivity analysis using only patients alive at 24 hours after ICU admission; more detail can be found in eTable 5 in the Supplement. Analyses were performed using Python (Python Software Foundation) with the scikit-learn package^27^ and R version 3.4.3 (R Foundation for Statistical Computing).

### Statistical Analysis

Data were analyzed from July 1, 2017, through August 1, 2018. All comparisons between models were based on 95% CIs, which correspond to a significance level of .05. A model was judged to be statistically significantly better performing compared with another if its 95% CI excluded the point estimate of the other model, and vice versa. These 95% CIs were formed by bootstrapping the results of 100 repetitions of nested 10-fold cross-validation, which yielded 1000 AUC values for each model. Unpaired *t* tests were also used to obtain 2-tailed *P* values based on these AUC values for each model, where applicable; in this case, the significance level was also taken to be .05. To assess the association of derived measures of clinical trajectory with mortality, we also used unpaired *t *tests and, where applicable, Wilcoxon rank sum tests.

## Results

We extracted data for the first ICU admission of 101 196 unique patients. Mean (SD) age was 61.3 (17.1) years; 51.3% of patients were male (n = 51 899) and 48.7% were female (n = 49 297). In-hospital mortality was 10.4% (n = 10 505) (Table 1 and eTable 3 in the Supplement); 14.7% of all deceased patients died in the first 24 hours after ICU admission.

**Table 1.** Characteristics of the Cohort

Characteristic	Value (N = 101 196)
Deaths, No. (%)	10 505 (10.4)
Length of stay, mean (SD) [IQR], d	
First ICU	3.5 (4.4) [1-3]
Hospital	11.6 (17.1) [4-13]
Age, mean (SD) [IQR], y	61.3 (17.1) [51-74]
Male, No. (%)	51 899 (51.3)
Age categories, No. (%)	
<40 y	12 197 (12.1)
40-59 y	30 567 (30.2)
60-79 y	42 828 (42.3)
>79 y	15 604 (15.4)
Type of ICU at first admission, No. (%)	
Combined medical and surgical	32 218 (31.8)
Medical	19 110 (18.9)
Surgical	21 910 (21.6)
Neurologic	14 242 (14.1)
Coronary care	13 716 (13.6)

Abbreviations: ICU, intensive care unit; IQR, interquartile range.

Across all patients, we retrieved a total of approximately 500 million data points associated with the types of laboratory test results and vital sign measurements recorded in the EHR within the first 24 hours after ICU admission. Of these data points, the baseline model used only approximately 5 million, or 1%, but the more complex models used all of them. The baseline models used 48 predictor variables, whereas the clinical trajectory–augmented models used 192, and those further augmented with NLP used 1192. Missingness rates in our data were generally low, except for measurements associated with arterial blood gas and lactate levels, and resulted in similar patterns across the 3 sites (eTable 4 in the Supplement).

### Model Performance

Across all sites, we found that enriching models with NLP-derived terms, variables measuring clinical trajectory, or both uniformly improved model discrimination, even in the worst case when models were trained using data from a single site and then tested on another (Table 2). Models trained on data from one teaching hospital and tested on data from the other exhibited the best performance (AUC for UCSF to BIDMC, 0.923; AUC for BIDMC to UCSF, 0.897), although performance remained good for models trained and tested with MPMC data, with AUCs of 0.894 for UCSF to MPMC and 0.854 for BIDMC to MPMC (Table 2). This finding demonstrates the external validity and portability of models incorporating these variables, even among different types of hospitals (teaching vs community) where documentation patterns and case mix may vary substantially.

**Table 2.** External Validation of Models Built on Each Participating Site

Participating Site	Type of Model by Test Site, AUC^a^								
	Baseline Model^b^			Clinical Trajectory–Augmented Model^c^			NLP-Augmented Model^d^		
	UCSF	MPMC	BIDMC	UCSF	MPMC	BIDMC	UCSF	MPMC	BIDMC
UCSF	NA	0.604	0.838	NA	0.801	0.876	NA	0.878	0.897
MPMC	0.781	NA	0.714	0.823	NA	0.803	0.894	NA	0.854
BIDMC	0.867	0.729	NA	0.888	0.814	NA	0.923	0.857	NA

Abbreviations: AUC, area under the receiver operating characteristic curve; BIDMC, Beth Israel Deaconess Medical Center; MPMC, Mills-Peninsula Medical Center; NA, not applicable; NLP, natural language processing; UCSF, University of California, San Francisco.

^a^ Calculated using nested 10-fold cross-validation. All comparisons of the AUCs for each train and test pair between models (eg, trained on BIDMC, tested at UCSF for model 1 vs model 2: 0.867 vs 0.888) were statistically significant at *P* < .05.

^b^ Uses the highest and lowest of all laboratory values and vital signs.

^c^ Adds measures of distribution, variability, and trajectory of laboratory values and vital signs to models already using the highest and lowest values.

^d^ Adds NLP to models already using all observed values and measures of distribution, variability, and trajectory of laboratory values and vital signs.

Furthermore, to obtain estimates of performance that most closely correspond to the real-world use of these models, we pooled data from all 3 sites to cross-validate a new set of models, adding types of predictive variables in an incremental fashion. First, the baseline model using only the highest and lowest observed values for each laboratory test result and vital sign achieved a cross-validated AUC of 0.831 (95% CI, 0.830-0.832) (Table 3). Augmenting this model with measures of clinical trajectory improved discrimination, as reflected by an increase in AUC to 0.899 (95% CI, 0.896-0.902; *P* < .001 for AUC difference). Finally, further enriching this model with NLP of clinical text increased the AUC to 0.922 (95% CI, 0.916-0.924; *P* < .001). These NLP-derived terms were associated with improved model performance even when applied across sites (AUC difference for UCSF: 0.077 to 0.021; AUC difference for MPMC: 0.071 to 0.051; AUC difference for BIDMC: 0.035 to 0.043; *P* < .001) when augmenting with NLP at each site. The gains in AUC at each step were similar to those observed in a sensitivity analysis that revalidated each of these 3 models in a separate cohort that included only patients alive at 24 hours, implying that the models are insensitive to measurements recorded immediately before death for patients who died before 24 hours (eTable 5 in the Supplement).

**Table 3.** Model Discrimination for Multicenter Models Using Different Data and Analytic Methods

Modeling Approach	AUC (95% CI)^a^
Using highest and lowest of all laboratory values and vital signs, logistic regression (baseline)	0.831 (0.830-0.832)
Adding information from all observed laboratory values and vital signs^b^	0.899 (0.896-0.902)
Adding NLP of clinical text^c^	0.922 (0.916-0.924)

Abbreviations: AUC, area under the receiver operating characteristic curve; NLP, natural language processing.

^a^ Calculated using nested 10-fold cross-validation; 95% CIs were computed using bootstrapping.

^b^ Adds measures of distribution, variability, and trajectory of laboratory values and vital signs to models already using the highest and lowest values.

^c^ Adds NLP to models already using all observed values and measures of distribution, variability, and trajectory of laboratory values and vital signs.

The AUPRCs were 0.265 (95% CI, 0.258-0.272) for the baseline model, 0.434 (95% CI, 0.412-0.456) for the clinical trajectory–augmented model, and 0.545 (95% CI, 0.532-0.568) for the clinical trajectory model when augmented with NLP-derived terms. All 3 model AUPRCs were significantly better than 0.10, which represents the prevalence of the mortality outcome in our sample and thus the AUPRC value that would have been obtained by chance. At the optimal cut point value, the sensitivity (recall) and positive predictive value (precision) were 0.623 and 0.312, respectively, for the baseline model, 0.828 and 0.429, respectively, for the clinical trajectory–augmented model, and 0.941 and 0.573, respectively, for the clinical trajectory model when augmented with NLP-derived terms. Finally, all models also had nonsignificant modified Hosmer-Lemeshow statistics (*C* = 12.1, *C* = 14.3, and *C* = 15.7, respectively; *P* > .05), suggesting good calibration, which was confirmed by examination of the calibration curves (eFigure 2 in the Supplement). The mortality rate among patients in the top decile of predicted mortality, based on the pooled model, was 92.3%.

### Exploration of Clinical Trajectory and Free-Text Predictors: Construct Validity

The models including the derived measures of clinical trajectory (eTable 6 in the Supplement) appeared to exhibit good construct validity. For instance, we observed that a positive linear trend (improvement) in a Glasgow Coma Scale score was independently associated with reduced mortality risk (mean trend for survivors vs nonsurvivors, 0.124 vs −0.034 points/h; *P* < .001). The same pattern also held for improvements in individual Glasgow Coma Scale components of eye response (mean trend for survivors vs nonsurvivors, 0.031 vs −0.012 points/h; *P* < .001), verbal response (mean trend for survivors vs nonsurvivors, 0.049 vs −0.016 points/h; *P* < .001), and to a lesser extent, motor response (mean trend for survivors vs nonsurvivors, 0.043 vs −0.002 points/h; *P* = .04). Increasing levels of bilirubin (mean difference between last and first recorded values for survivors vs nonsurvivors, −0.035 vs 0.124 mg/dL [to convert to μmol/L, multiply by 17.104]; *P* < .001), urea (mean difference between last and first recorded values for survivors vs nonsurvivors, −0.657 vs 0.308 mg/dL [to convert to mmol/L, multiply by 0.357]; *P* < .001), sodium (mean difference between last and first recorded values for survivors vs nonsurvivors, 0.345 vs 0.990 mEq/L [to convert to mmol/L, multiply by 1.0]; *P* < .001), potassium (mean difference between last and first recorded values for survivors vs nonsurvivors, −0.074 vs 0.099 mEq/L [to convert to mmol/L, multiply by 1.0]; *P* = .002), and lactate (mean difference between last and first recorded values for survivors vs nonsurvivors, −0.387 vs 0.802 mg/dL [to convert to mmol/L, multiply by 0.111]; *P* = .006), as measured by the differences between first and last values within the first 24 hours after ICU admission, were each independently associated with increased mortality risk.

Models incorporating clinical free-text terms as predictors also demonstrated good construct validity. Terms suggesting acutely decompensated states (*sepsis*, *shock*, and *coagulopathy*), the use of emergent interventions (*ECMO* or *CVVH* [continuous venovenous hemofiltration]), or physical examination signs portending a poor prognosis (*pupils fixed*, *gag* [as in gag reflex], and *ascites*) were most strongly associated with mortality (Table 4). Terms associated with increased survival included those indicating surgical status (*EBL* [estimated blood loss], *POD* [postoperative day], and *OHNS* [otolaryngology–head and neck surgery]), as well as physical examination findings associated with normal neurologic examination findings (*denies* [as in, eg, denies pain], *awake*, or *alert*) and extubation (eg, *extubated*) (Table 4). We found in preliminary experiments that using 2-word phrases did not appear to improve prediction over the use of single words, although some 2-word phrases could include negations (eg, *not septic*). Among the lists of terms extracted for use at each site, we did not find any that appeared to indicate the event of death or planning for death, for example, *expired* or *CMO*.

**Table 4.** Examples of Influential Predictive Terms Derived From Clinical Text

Clinical Term	Weight^a^
Pupils (fixed)	7.78
Gag	6.74
ECMO	6.18
Coagulopathy	4.67
Shock	4.41
Intubated	4.28
PEA	3.68
Chemotherapy	3.49
Ascites	3.27
CVVH	2.78
Sepsis	2.27
Meropenem	2.09
EtOH	−1.14
OHNS	−1.15
Alert	−1.51
EBL	−2.10
Diet	−2.68
Awake	−3.11
PERRL	−4.28
Denies (pain)	−4.56
POD	−4.70
Extubated	−7.64

Abbreviations: CVVH, continuous venovenous hemofiltration; EBL, expected blood loss; ECMO, extracorporeal membrane oxygenation; EtOH, ethanol (alcohol); OHNS, otolaryngology–head and neck surgery; PEA, pulseless electrical activity; PERRL, pupils equal, round, and reactive to light; POD, postoperative day.

^a^ Each term is associated with a β coefficient or weight in the logistic regression model, which represents its relative association with mortality. Positive weights indicate increased odds of mortality when the term is included in a clinical note. Negative weights indicate decreased odds of mortality.

## Discussion

We report the development and validation of 2 generalizable modeling approaches that predict in-hospital mortality well using the first 24 hours of data after ICU admission. Leveraging newly available computational power and EHR data enables models to be augmented with measures of clinical trajectory and NLP-derived terms, which yield the observed gains in predictive performance. The resulting models appeared to maintain good construct and external validity, despite a varied case mix derived from academic and community hospitals. Moreover, these approaches can be easily implemented using open-source machine learning tools. Notably, our approach is distinct from previous work primarily in that we assess the generalizability of these 2 modeling approaches, particularly that of using unstructured clinical free text, which, to our knowledge, has not been validated across institutions.

Our best-performing model achieved an AUC of 0.922 compared with 0.88 reported for APACHE IV,^2^ 0.85 for the Simplified Acute Physiology Score III,^15,16^ 0.82 for the Mortality Probability Admission Model III,^13^ 0.85 for physician predictions in a meta-analysis,^28^ and 0.67 for a recent study by Detsky et al.^29^ Although we were not able to compare our models directly with these approaches on the same patients, augmenting our base model with measures of clinical trajectory and NLP terms appeared to significantly improve discrimination. Although all models used the same laboratory test results and vital signs as data sources for predictive variables, the baseline models took advantage of only approximately 1% of the data points available in EHRs, whereas our clinical trajectory– and NLP-augmented models used all such data points.

Notably, our models incorporating NLP took advantage of unstructured clinical free text, which represents a novel data source for risk models. To our knowledge, this is the first study of ICU risk adjustment to integrate, from multiple hospital systems’ EHRs, variables derived from structured data (laboratory test results and vital signs) and clinical text into a single model and to assess the generalizability of such models across institutions. Although for example, clinical free text alone has previously been used to predict outcomes,^10,30^ for case finding and registry construction,^31,32^ or for information retrieval from EHRs,^33,34,35^ it has not been validated across different institutions to facilitate ICU risk modeling.

Recently, Weissman et al^36^ studied the feasibility of incorporating clinical free text into a model to predict the combined outcome of mortality or prolonged length of stay, but their analysis was limited to a single institution, so they were not able to assess generalizability. Moreover, Weissman et al^36^ found only very small marginal gains in predictive performance when using more complex machine learning methods, namely gradient boosting, over regularized logistic regression, as we used here.

Rajkomar et al^37^ developed models incorporating notes to predict in-hospital mortality and length of stay. However, their study included all inpatients, not just patients in the ICU, and only assessed model performance within, and not across, each institution in their study, leaving open the question of the generalizability of their approach. Moreover, their approach extracts predictive variables from outpatient and other notes not associated with the hospital stay, which has the potential to introduce bias related to data availability, possibly limiting generalizability.

Recently, Delahanty et al^38^ also built a model to predict ICU mortality from a multi-institutional sample. However, they used not just data available during the first 24 hours, but also diagnosis-related group and cost-weight data from claims, and in fact claims-based variables had the greatest predictive power in their final model.

Finally, Badawi et al^39^ also used a multi-institutional ICU data set to develop a similar model. However, their primary goal was to validate serially computed risk scores throughout a patient’s ICU stay using data from within the 24 hours before death, not to develop an on-admission risk model. Furthermore, their approach did not validate predictive variables derived from clinical free text.

### Limitations

Our study has important limitations. We were not able to directly compare our models with, for example, APACHE IV, owing to the cost of data collection required for a cohort of our size. Instead, to approximate those models, we developed a surrogate baseline model using minimum and maximum values of each predictor. It exhibited discrimination comparable to the Simplified Acute Physiology Score III and Mortality Probability Admission Model III and fell slightly below the values reported for APACHE IV in the literature. Second, we validated our models using data from only 3 institutions with 20 ICUs, but our sample size of 101 196 patients is similar in magnitude to those in previous model validation studies.^2,8^ Third, we found some variation in model performance improvements between sites, particularly when data from MPMC were used for training and testing.

Moreover, because our models from each site used only the 1000 most common terms appearing in notes at that site, we were able to determine, by inspection of these terms, that none were protected health information, such as patient names. Thus, in this instance, simply limiting the models to the most common terms achieved complete deidentification. Further research would be needed to confirm whether this finding is typical of text at other institutions and whether more terms could be used while maintaining generalizability and ensuring privacy.

Although NLP-augmented models appear to generalize well, even between academic and community settings, their generalizability to any one hospital may not be guaranteed, particularly if not validated externally. Models using NLP, while potentially more accurate, may also be susceptible to being gamed by unscrupulous heath care professionals who construct notes in such a way to inflate predicted mortality risks for their patients. As such models become more widely disseminated, further research will be needed to characterize the extent of these gaming behaviors and to develop mitigation strategies, including periodic audits and model recalibration.

## Conclusions

Compared with existing methods using only the single most abnormal laboratory test results and vital signs from the first 24 hours after ICU admission, trends of severity of illness in the ICU can be quantified, and mortality thus more accurately predicted, by analyzing all the data available in the EHR and by incorporating information readily extracted from text notes. Clinical trajectory and NLP models built using these methods can be adapted to EHRs for use by health care professionals and researchers for a variety of purposes, including risk adjustment in clinical studies and quality improvement initiatives.

## Notes

Supplement. **eFigure 1.** Three Clinical Scenarios That Demonstrate the Need for More Sophisticated Analyses of ICU Physiological Data **eFigure 2.** Calibration of the 2 Models Validated on Data From All 3 Institutions Using 10-fold Cross Validation **eTable 1.** Laboratory Test Results and Vital Signs **eTable 2.** Derived Measures of Variability and Clinical Trajectory for Each Laboratory Test Result and Vital Sign **eTable 3.** Characteristics of the Study Population by Site **eTable 4.** Rates of Missingness Among the Predictive Variables Used in Our Analysis **eTable 5.** Results of the Sensitivity Analysis Comparing Validation Using All Patients With Using Only Those Alive at 24 Hours **eTable 6.** List of Coefficient Values of Derived Measures of Clinical Trajectory for the Pooled Model (Model 2 in the Text) With 192 Such Variables
