# Supplementary material for: Validation of Prediction Models for Critical Care Outcomes Using Natural Language Processing of Electronic Health Record Data
Source: JAMA Netw Open. 2018 Dec 21;1(8):e185097. doi: 10.1001/jamanetworkopen.2018.5097 (PMC6324323; doi:10.1001/jamanetworkopen.2018.5097)
Supplement: Supplement. — eFigure 1. Three Clinical Scenarios That Demonstrate the Need for More Sophisticated Analyses of ICU Physiological Data eFigure 2. Calibration of the 2 Models Validated on Data From All 3 Institutions Using 10-fold Cross Validation eTable 1. Laboratory Test Results and Vital Signs eTable 2. Derived Measures of Variability and Clinical Trajectory for Each Laboratory Test Result and Vital Sign eTable 3. Characteristics of the Study Population by Site eTable 4. Rates of Missingness Among the Predictive Variables Used in Our Analysis eTable 5. Results of the Sensitivity Analysis Comparing Validation Using All Patients With Using Only Those Alive at 24 Hours eTable 6. List of Coefficient Values of Derived Measures of Clinical Trajectory for the Pooled Model (Model 2 in the Text) With 192 Such Variables [file jamanetwopen-1-e185097-s001.pdf]

Supplementary Online Content

Marafino BJ, Park M, Davies JM, et al. Validation of prediction models for critical care outcomes using natural language processing of electronic health record data. *JAMA Netw Open*. 2018;1(8):e185097. doi:10.1001/jamanetworkopen.2018.5097

eFigure 1. Three Clinical Scenarios That Demonstrate the Need for More Sophisticated Analyses of ICU Physiological Data
eFigure 2. Calibration of the 2 Models Validated on Data From All 3 Institutions Using 10-fold Cross Validation
eTable 1. Laboratory Test Results and Vital Signs
eTable 2. Derived Measures of Variability and Clinical Trajectory for Each Laboratory Test Result and Vital Sign
eTable 3. Characteristics of the Study Population by Site
eTable 4. Rates of Missingness Among the Predictive Variables Used in Our Analysis
eTable 5. Results of the Sensitivity Analysis Comparing Validation Using All Patients With Using Only Those Alive at 24 Hours
eTable 6. List of Coefficient Values of Derived Measures of Clinical Trajectory for the Pooled Model (Model 2 in the Text) With 192 Such Variables

This supplementary material has been provided by the authors to give readers additional information about their work.

© 2018 Marafino BJ et al. *JAMA Network Open.*

eFigure 1. Three Clinical Scenarios That Demonstrate the Need for More Sophisticated Analyses of ICU Physiological Data

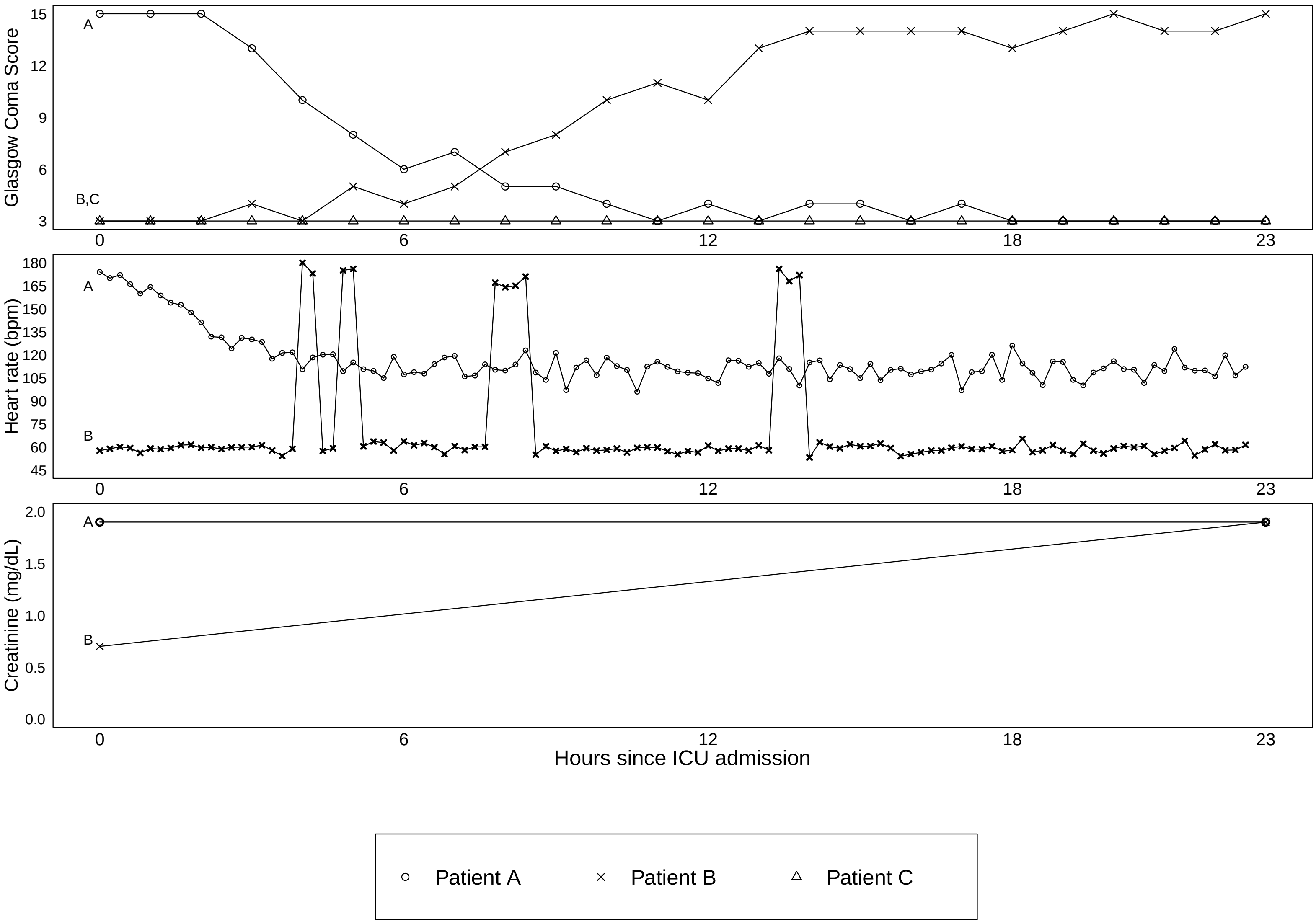

In Panel **a)**, all three patients–A, B, and C–are recorded as having the same single worst value (SWV) of 3 of the Glasgow Coma Score (GCS) over the 24-hour period following ICU admission. Patient A is admitted with a GCS value of 15, but his neurological status deteriorates rapidly (e.g. as in the case of an epidural hematoma), resulting in a GCS of 3. Patient B follows the opposite course, being admitted with a GCS of 3 before improving to a GCS of 15. Patient C is admitted with

© 2018 Marafino BJ et al. *JAMA Network Open.*

a GCS of 3, but never improves. In contrast, using the slopes of the linear trends associated with each of these three series of GCS could stratify the mortality risk.

Similarly, Panel **b)** depicts the time course of heart rate (HR) for two patients admitted to the ICU following myocardial infarction. Patient A experiences atrial fibrillation with rapid ventricular response, which is managed with β-blockers to achieve a target rate of 110 bpm. Patient B, meanwhile, experiences intermittent ventricular tachycardia, marked by periods of elevated HR >160 bpm before returning to a relatively stable baseline. Both patients are recorded as having a SWV of HR roughly in the same range, resulting in similar risk estimates. However, the persistent variability (even though rate control is achieved) of Patient A's HR, as indicated by the standard deviation of the series, may indicate a poorer prognosis compared to Patient B.

Panel **c)** depicts serum creatinine (SCr) values over time for two patients. Two measurements of SCr are obtained for each patient. Patient A is initially admitted to the ICU with comorbid chronic kidney disease following surgery. His SCr on admission is 1.9mg/dL, and a repeat measurement 24 hours later is unchanged. However, Patient B experiences an rapid elevation of SCr from 0.7 to 1.9mg/dL in the 24 hours following admission, indicating the onset of acute kidney injury caused by, e.g., renal hypoperfusion associated with septic shock. Both patients attain the same SWV of SCr, even though Patient B has a poorer prognosis, as indicated by using the difference between first and last measurements within this 24-hour window.

© 2018 Marafino BJ et al. *JAMA Network Open.*

eFigure 2. Calibration of the 2 Models Validated on Data From All 3 Institutions Using 10-fold Cross Validation

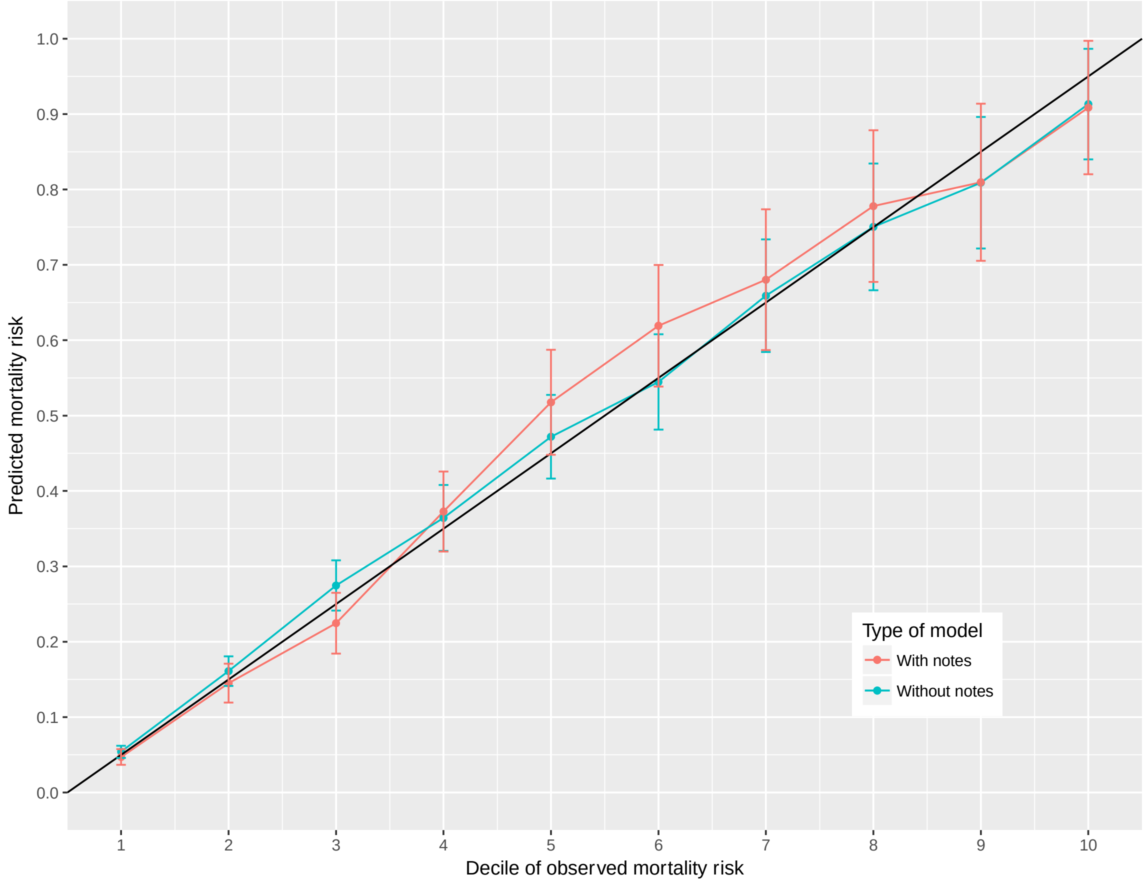

Includes models 2 and 3 in Methods. The error bars depict the bootstrap 95% confidence intervals. Note that the model with notes tends to underpredict mortality for the lower-risk deciles as compared to the model without notes, and vice versa for patients in the middle deciles of risk, but these differences do not appear significant.

© 2018 Marafino BJ et al. *JAMA Network Open.*

eTable 1. Laboratory Test Results and Vital Signs

**Laboratory values**
Arterial blood gas components
Bicarbonate (mmol/L)
$PaCO_2$ (mm Hg)
pH
PaO_2 (mm Hg)
Blood count components
Hematocrit (%)
Platelets (x10^9/L)
White blood cells (x10^9/L)
Other chemistries
Bilirubin (mg/dL)
Creatinine (mg/dL)
Glucose (mg/dL)
Lactate (mmol/L)
Potassium (mmol/L)
Sodium (mmol/L)
Urea (mg/dL)
**Vital signs**
Temperature (degrees Celsius)
Heart rate (beats per minute)
Respiratory rate (breaths per minute)
Oxygen saturation (%)
Mean arterial pressure (mm Hg)
Fraction of inspired oxygen (FiO_2) (%)
Glasgow Coma Scale
Total score (3-15)
Eye response (1-4)
Verbal response (1-6)
Motor response (1-5)

All observations in the first 24 hours of ICU admission were retrieved from the electronic health record.

© 2018 Marafino BJ et al. *JAMA Network Open.*

eTable 2. Derived Measures of Variability and Clinical Trajectory for Each Laboratory Test Result and Vital Sign

**Measures of the distribution of each laboratory test or vital sign**
First value observed within the 24 hours following ICU admission
Last value observed within the 24 hours following ICU admission
Maximum value observed within the 24 hours following ICU admission
Minimum value observed within the 24 hours following ICU admission
Mean
**Measures of variability or clinical trajectory**
Standard deviation
First value minus last value collected
Linear trend as fit by least squares

© 2018 Marafino BJ et al. *JAMA Network Open.*

eTable 3. Characteristics of the Study Population by Site

	MPMC	UCSF	BIDMC
Patients, number	15,666	38,624	46,906
Deaths, number (%)	1,770 (11.3%)	3,581 (9.3%)	5,154 (11.0%)
Length of first ICU stay, mean (IQR), days	2.2 (0-2)	3.3 (1-3)	4.1 (1-4)
Length of hospital stay, mean (IQR), days	8.5 (3-10)	14.6 (4-16)	10.1 (4-12)
Age, mean ± SD (IQR), y	68.0±16.6 (57-81)	57.5±17.1 (47-69)	62.1±16.5 (51-76)
Male gender, number (%)	6,993 (44.6%)	18,056 (46.7%)	26,850 (57.2%)
Age categories, y			
<40	978 (6.2%)	6,291 (16.3%)	4,928 (10.5%)
40-59	3,569 (22.8%)	13,080 (33.9%)	13,918 (29.7%)
60-79	6,559 (41.9%)	15,755 (40.8%)	20,514 (43.7%)
>79	4,560 (29.1%)	3,498 (9.1%)	7,546 (16.1%)
Type of ICU at first admission			
Combined medical/surgical	15,666 (100%)	16,552 (42.8%)	0 (0%)
Medical	0 (0%)	558 (1.4%)	18,552 (39.6%)
Surgical	0 (0%)	0 (0%)	21,910 (46.7%)
Neurological	0 (0%)	14,242 (36.9%)	0 (0%)
Coronary care	0 (0%)	7,272 (18.9%)	6,444 (13.7%)
Abbreviations: MPMC: Mills-Peninsula Medical Center; UCSF: University of California, San Francisco; BIDMC: Beth Israel Deaconess Medical Center.			

© 2018 Marafino BJ et al. *JAMA Network Open.*

eTable 4. Rates of Missingness Among the Predictive Variables Used in Our Analysis

Variable	Missingness, %		
	MPMC	UCSF	BIDMC
Lactate	53.49	43.82	40.19
Urea	0.86	3.09	0.25
Creatinine	1.93	1.44	0.23
Hematocrit	1.29	1.00	0.12
Sodium	1.45	0.83	0.19
Potassium	1.37	0.86	0.10
PCO2	57.99	28.50	33.70
Bilirubin	42.97	54.60	54.33
Glucose	1.04	5.85	0.20
Platelets	1.55	3.09	0.45
PO2	51.33	27.63	33.70
WBC	1.55	1.18	0.66
pH	52.05	27.63	30.85
Bicarbonate	53.95	0.76	33.70
MAP	0.00	0.03	0.33
Temperature	0.00	0.02	0.41
Respirations	0.00	0.01	0.41

© 2018 Marafino BJ et al. *JAMA Network Open.*

eTable 5. Results of the Sensitivity Analysis Comparing Validation Using All Patients With Using Only Those Alive at 24 Hours

	**All patients**	**Only patients alive at 24 hours**	**Difference**
Base model, best and worst coded values only	.883	.866	*-.017*
Add all coded values	.898	.882	*-.016*
Increment in AUC	**+.015**	**+.016**	***+.001***
Add notes	.913	.899	*-.016*
Increment in AUC	**+.015**	**+.017**	***+.002***

To assess whether the models were unduly influenced by data from patients who died within the 24-hour period for which we collected data, we conducted a sensitivity analysis by developing models using only patients alive at 24 hours following ICU admission. We re-validated the models in this separate cohort and compared our results to those obtained in the original cohort. For each model, we compared the difference in AUC increases observed for each cohort relative to that of the model validated in the previous step – i.e., the difference in AUC increase for model #2 as compared to model #1 (baseline single worst value model), and similarly, model #3 (model #2 + NLP) was compared to model #2. The incremental gains in AUC as additional data is made available to the predictive algorithms when including patients who died in the first 24 hours were very similar to the incremental gains when excluding such patients.

© 2018 Marafino BJ et al. *JAMA Network Open.*

eTable 6. List of Coefficient Values of Derived Measures of Clinical Trajectory for the Pooled Model (Model 2 in the Text) With 192 Such Variables

Derived measure of clinical trajectory	Coefficient value
Lactate, maximum value	3.76
Lactate, minimum value	5.78
Lactate, mean	3.54
Lactate, standard deviation	2.22
Lactate, coefficient for linear trend	0.66
Lactate, first value collected	2.39
Lactate, last value collected	8.41
Lactate, last minus first value	4.33
Urea, maximum value	4.67
Urea, minimum value	5.34
Urea, mean	5.06
Urea, standard deviation	1.07
Urea, coefficient for linear trend	1.06
Urea, first value collected	4.34
Urea, last value collected	6.07
Urea, last minus first value	2.92
Creatinine, maximum value	-0.57
Creatinine, minimum value	0.05
Creatinine, mean	-0.20
Creatinine, standard deviation	-2.07
Creatinine, coefficient for linear trend	1.85
Creatinine, first value collected	-0.69
Creatinine, last value collected	0.48
Creatinine, last minus first value	3.17
Hematocrit, maximum value	-1.86
Hematocrit, minimum value	-0.53
Hematocrit, mean	-1.22
Hematocrit, standard deviation	-1.34
Hematocrit, coefficient for linear trend	-0.34
Hematocrit, first value collected	-1.58
Hematocrit, last value collected	-0.37
Hematocrit, last minus first value	2.02
Sodium, maximum value	-1.11
Sodium, minimum value	-2.45

© 2018 Marafino BJ et al. *JAMA Network Open.*

Sodium, mean	-3.02
Sodium, standard deviation	4.56
Sodium, coefficient for linear trend	0.55
Sodium, first value collected	-3.07
Sodium, last value collected	-0.24
Sodium, last minus first value	3.55
Potassium, maximum value	-0.04
Potassium, minimum value	0.93
Potassium, mean	0.51
Potassium, standard deviation	0.31
Potassium, coefficient for linear trend	2.30
Potassium, first value collected	0.39
Potassium, last value collected	0.80
Potassium, last minus first value	0.18
PaO2, maximum value	-4.08
PaO2, minimum value	-3.46
PaO2, mean	-4.64
PaO2, standard deviation	0.40
PaO2, coefficient for linear trend	0.64
PaO2, first value collected	-4.08
PaO2, last value collected	-2.24
PaO2, last minus first value	1.79
Bilirubin, maximum value	3.72
Bilirubin, minimum value	4.29
Bilirubin, mean	4.09
Bilirubin, standard deviation	0.76
Bilirubin, coefficient for linear trend	1.49
Bilirubin, first value collected	3.46
Bilirubin, last value collected	4.52
Bilirubin, last minus first value	3.55
Glucose, maximum value	-1.21
Glucose, minimum value	0.82
Glucose, mean	-0.22
Glucose, standard deviation	1.19
Glucose, coefficient for linear trend	-0.55
Glucose, first value collected	1.02
Glucose, last value collected	1.89
Glucose, last minus first value	0.07
PaCO2, maximum value	1.77

© 2018 Marafino BJ et al. *JAMA Network Open.*

PaCO2, minimum value	-1.37
PaCO2, mean	-1.31
PaCO2, standard deviation	0.04
PaCO2, coefficient for linear trend	-1.05
PaCO2, first value collected	-0.20
PaCO2, last value collected	-5.25
PaCO2, last minus first value	-5.95
Platelets, maximum value	-0.60
Platelets, minimum value	-0.91
Platelets, mean	-0.18
Platelets, standard deviation	1.34
Platelets, coefficient for linear trend	-0.02
Platelets, first value collected	-2.65
Platelets, last value collected	0.78
Platelets, last minus first value	7.41
White blood cells, maximum value	0.18
White blood cells, minimum value	2.10
White blood cells, mean	0.70
White blood cells, standard deviation	-2.74
White blood cells, coefficient for linear trend	-1.32
White blood cells, first value collected	1.08
White blood cells, last value collected	1.08
White blood cells, last minus first value	-0.21
pH, maximum value	1.43
pH, minimum value	0.03
pH, mean	1.70
pH, standard deviation	0.73
pH, coefficient for linear trend	0.19
pH, first value collected	1.56
pH, last value collected	-4.29
pH, last minus first value	-4.46
Bicarbonate, maximum value	0.69
Bicarbonate, minimum value	0.72
Bicarbonate, mean	1.27
Bicarbonate, standard deviation	-0.24
Bicarbonate, coefficient for linear trend	-0.41
Bicarbonate, first value collected	0.87
Bicarbonate, last value collected	-2.26
Bicarbonate, last minus first value	-5.28

© 2018 Marafino BJ et al. *JAMA Network Open.*

Mean arterial pressure, maximum value	-0.70
Mean arterial pressure, minimum value	-2.45
Mean arterial pressure, mean	-3.85
Mean arterial pressure, standard deviation	-2.63
Mean arterial pressure, coefficient for linear trend	0.03
Mean arterial pressure, first value collected	-0.16
Mean arterial pressure, last value collected	-5.93
Mean arterial pressure, last minus first value	-4.25
Temperature, maximum value	-0.93
Temperature, minimum value	-1.64
Temperature, mean	-3.80
Temperature, standard deviation	1.14
Temperature, coefficient for linear trend	1.07
Temperature, first value collected	-1.89
Temperature, last value collected	-1.84
Temperature, last minus first value	0.23
FiO2, maximum value	-1.25
FiO2, minimum value	7.05
FiO2, mean	7.81
FiO2, standard deviation	1.43
FiO2, coefficient for linear trend	-0.81
FiO2, first value collected	-1.73
FiO2, last value collected	6.46
FiO2, last minus first value	-0.12
RESP, maximum value	2.37
RESP, minimum value	5.54
RESP, mean	3.15
RESP, standard deviation	-1.51
RESP, coefficient for linear trend	-0.84
RESP, first value collected	3.49
RESP, last value collected	1.73
RESP, last minus first value	-1.62
Glasgow Coma Scale (GCS), maximum value	-6.38
Glasgow Coma Scale (GCS), minimum value	-2.51
Glasgow Coma Scale (GCS), mean	-5.06
Glasgow Coma Scale (GCS), standard deviation	-2.50
Glasgow Coma Scale (GCS), coefficient for linear trend	-0.83
Glasgow Coma Scale (GCS), first value collected	-1.82
Glasgow Coma Scale (GCS), last value collected	-7.09

© 2018 Marafino BJ et al. *JAMA Network Open.*

Glasgow Coma Scale (GCS), last minus first value	-4.49
GCS eye response, maximum value	-6.61
GCS eye response, minimum value	-1.24
GCS eye response, mean	-5.07
GCS eye response, standard deviation	-2.71
GCS eye response, coefficient for linear trend	-1.65
GCS eye response, first value collected	-1.47
GCS eye response, last value collected	-7.18
GCS eye response, last minus first value	-4.43
Pulse, maximum value	6.02
Pulse, minimum value	2.94
Pulse, mean	8.16
Pulse, standard deviation	2.50
Pulse, coefficient for linear trend	2.02
Pulse, first value collected	2.49
Pulse, last value collected	3.68
Pulse, last minus first value	0.92
GCS motor response, maximum value	-4.93
GCS motor response, minimum value	-3.11
GCS motor response, mean	-4.89
GCS motor response, standard deviation	-0.95
GCS motor response, coefficient for linear trend	1.27
GCS motor response, first value collected	-2.22
GCS motor response, last value collected	-5.72
GCS motor response, last minus first value	-2.07
GCS verbal response, maximum value	-5.41
GCS verbal response, minimum value	-2.02
GCS verbal response, mean	-4.23
GCS verbal response, standard deviation	-2.18
GCS verbal response, coefficient for linear trend	-2.49
GCS verbal response, first value collected	-1.08
GCS verbal response, last value collected	-6.42
GCS verbal response, last minus first value	-5.35
Oxygen saturation, maximum value	0.06
Oxygen saturation, minimum value	-1.91
Oxygen saturation, mean	-5.39
Oxygen saturation, standard deviation	4.00
Oxygen saturation, coefficient for linear trend	-4.03
Oxygen saturation, first value collected	-1.10

© 2018 Marafino BJ et al. *JAMA Network Open.*

Oxygen saturation, last value collected	-6.75
Oxygen saturation, last minus first value	-4.64

© 2018 Marafino BJ et al. *JAMA Network Open.*
